# Effect of Foot Reflexology on Pain, Fatigue, and Quality of Sleep after Kidney Transplantation Surgery: A Parallel Randomized Controlled Trial

**DOI:** 10.1155/2020/5095071

**Published:** 2020-08-01

**Authors:** Atena Samarehfekri, Mahlagha Dehghan, Mansoor Arab, Mohammad Reza Ebadzadeh

**Affiliations:** ^1^Nursing Research Center, Razi Faculty of Nursing and Midwifery, Kerman University of Medical Sciences, Kerman, Iran; ^2^Department of Critical Care Nursing, Razi Faculty of Nursing and Midwifery, Kerman University of Medical Sciences, Kerman, Iran; ^3^Faculty of Nursing and Midwifery, Bam University of Medical Sciences, Bam, Iran; ^4^Department of Urology, School of Medicine, Physiology Research Center, Neuropharmacology Research Institute, Shahid Bahonar Hospital, Kerman University of Medical Sciences, Kerman, Iran

## Abstract

**Materials and Methods:**

The study was a parallel randomized controlled trial. Patients admitted to the transplantation ward participated in the study. Fifty-three eligible patients were allocated into the foot reflexology group (*n* = 26) and the control group (*n* = 27) by using the stratified randomization method. Finally, 25 participants in each group finished the study. The intervention group received foot reflexology for 30 minutes once a day for three consecutive days, and no reflexology was applied in the control group. The intervention started on the second day after surgery. Pain, fatigue, and quality of sleep were measured on the first, second (before intervention), third, fourth, and eleventh days after surgery. Data were collected using visual analogue scale for measuring pain and fatigue and Verran and Snyder-Halpern sleep scale for measuring quality of sleep.

**Results:**

In each group, 25 patients finished the study. The mean pain score in the foot reflexology and control groups decreased from 9.44 ± 0.96 and 9.36 ± 0.91 on the day of surgery to 1.32 ± 0.94 and 4.32 ± 1.68 on the eleventh day after surgery, respectively. The mean fatigue score in the reflexology and control groups decreased from 8.76 ± 1.27 and 8.6 ± 1.26 on the day of surgery to 1.24 ± 1.2 and 3.92 ± 1.63 on the eleventh day after surgery, respectively. The mean sleep score in the foot reflexology and control groups increased from 33.38 ± 11.22 and 39.59 ± 12.8 on the day of surgery to 69.43 ± 12.8 and 56.27 ± 8.03 on the eleventh day after surgery, respectively. While pain, fatigue, and sleep quality scores improved in both groups, those in the intervention group showed significantly greater improvement compared with the control group (*P* < 0.001). No significant difference was found between the two groups in the use of acetaminophen on the first, second, third, fourth, and eleventh days after surgery (*P* > 0.05).

**Conclusion:**

Foot reflexology may reduce pain and fatigue and improve sleep quality of patients after kidney transplantation.

## 1. Introduction

Kidney transplantation is the most effective treatment for end-stage kidney disease worldwide [1]. According to the statistics from the Global Observatory on Donation and Transplantation, the cases of kidney transplantation were 90,306 worldwide in 2017 [2]. In Iran, 48.8% of kidney failure patients are undergoing kidney transplantation [3]. Evidence suggests that successful kidney transplantation can improve quality of life, life expectancy, and reduce health costs [4]. However, patients may experience different physical difficulties such as cardiovascular and neurologic complications [5], sexual dissatisfaction [6], or mental disorders such as anxiety, depression, or stress [7]. Patients may also experience postoperative pain, fatigue, and sleep disorders [8].

Some patients experience severe pain on their back, chest, inguinal area, the surgery area, and head after kidney transplantation surgery [9, 10], and postsurgical pain is a major therapeutic problem in these patients [11]. This pain may become worse if not managed properly [12]. An inverse association is available between pain and blood pressure, and uncontrolled postoperative pain leads to hypotension and other postoperative disorders [13]. Fatigue and lack of energy are other common postoperative symptoms [14]. Few studies have shown that patients under kidney transplantation experience more fatigue than healthy subjects [15]. The prevalence of postoperative fatigue was 48.3% in one study, which was 41.5% three months later and 38.1% six months later [16]. Fatigue can affect quality of sleep of kidney transplant recipients. On the other hand, sleep deprivation in these patients can cause fatigue, depression, pain, and stress [17, 18].

Different complementary and alternative medicine (CAM) methods such as foot reflexology are used for managing symptoms among some patients after kidney transplantation [19]. Foot reflexology is a special form of massage that accompanies with the pressure of the fingers, especially the thumbs on the reflex areas usually in the feet. These areas are believed to associate with all parts of the body, and applying pressure on them can affect the physiological responses of the body. They are thought to improve recovery and return homeostasis [20]. Foot reflexology can regulate blood circulation and hemodynamic variables [21]. The underlying mechanisms of reflexology are not well understood. Reflexology is assumed to facilitate relaxation, release endorphins, and modulate pain-impulse transmission and pain perception [22]. Subsequently, relaxation can effect quality of sleep and fatigue [23–25]. In addition, touch and massage of reflex points in the foot may reduce patients' pain. Diseases are caused by the blockage of energy in the body, and stimulation of reflex points may eliminate these obstructions and release energy in the body [26].

Several studies have examined the effects of foot reflexology on symptoms such as pain, fatigue, and quality of sleep of patients [19, 27–30]. Other studies showed a positive effect of foot reflexology on pain and anxiety of patients after general and spinal surgery [29, 30] and during chemotherapy and after breast cancer surgery [31, 32]. Different studies showed the positive effects of reflexology on alleviating fatigue in patients [33, 34]. Asltoghiri et al. showed the improvement of sleep disorders using reflexology [35]. Lee considered foot reflexology as a useful intervention to decrease fatigue and promote quality of sleep [36]. Moreover, the results of a systematic review showed that reflexology was safe and effective for insomnia, but further studies with greater accuracy and power are needed [37].

The complementary and alternative therapies have been increasingly used in recent decades, and nurses prefer to use noninvasive methods with minimal side effects [36]. Since reflexology does not have major side effects [37], nurses can use it to improve the quality of nursing care. However, decisions are still being made with caution due to insufficient research studies. No study has investigated the effect of reflexology on pain, fatigue, and quality of sleep after kidney transplantation; therefore, the current study tested the hypothesis that the mean scores of pain, fatigue, and quality of sleep in patients after kidney transplantation surgery were different between the foot reflexology and control groups after the intervention and one week later.

## 2. Materials and Methods

### 2.1. Study Type and Setting

This study was a parallel randomized controlled trial. Patients taken to the transplantation department of Afzalipour Hospital, Kerman, Iran, were studied from April 2018 to May 2019.

### 2.2. Sample Size and Sampling

**Figure 1:**
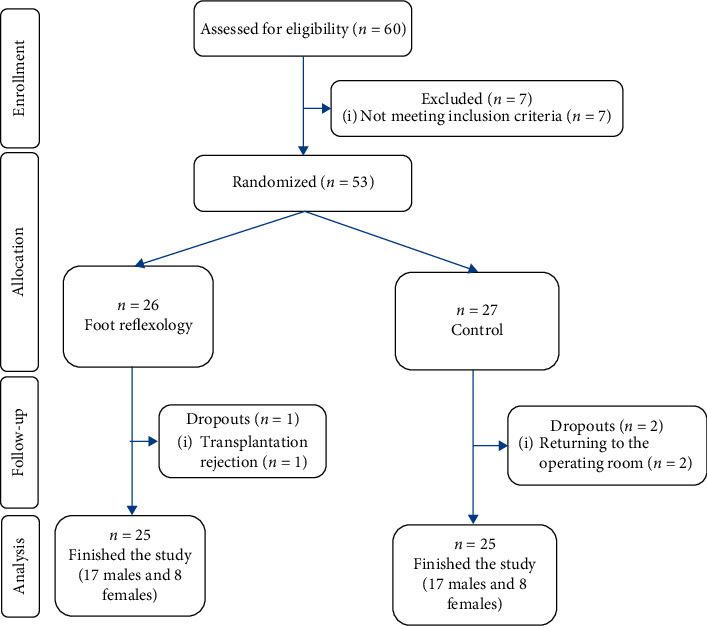
The flow diagram of the study.

According to a pilot study (5 samples in each group) (on the fourth day after surgery, the mean and standard deviation in the pilot reflexology group were 3.2 ± 2.17 and the mean and standard deviation in the pilot control group were 5.2 ± 1.3), the sample size was estimated to be 21 individuals for each group with a confidence coefficient of 95% and type II error of 10%. Due to the probability of dropout, 25 samples were selected in each group. It is noteworthy that the pilot samples were included in the final analysis. Furthermore, power analysis calculated with G^*∗*^Power software indicates that (power = 90%, *P*=0.05) 46 participants would be needed (23 per group) to detect an effect size of 0.2. Inclusion criteria were the minimum age of 15 years old, the first turn of the kidney transplantation, no ulcers or injuries in feet, especially the sole, complete postoperative consciousness, no history of using foot reflexology, no addiction to drug use or alcohol, and no mental disorder. Exclusion criteria were the patient's return to the operating room during the study, the patient's need to a sedative, and any symptoms indicative of transplant rejection (with the doctor's diagnosis). Sixty patients were examined for the inclusion criteria, of whom seven were not eligible due to different reasons such as a second transplant, no age fulfillment, and mental disorder. In addition, there were three dropouts due to returning to the operating room and rejection of the transplant. Finally, 25 samples in each group completed the study, and their data were analyzed (Figure 1). Eligible patients were selected by convenience sampling and allocated to the intervention and control groups with the stratified randomization method using gender and age (±2) as strata. In other words, the first sample was randomly allocated either to the intervention or control groups (using the lottery), and the subsequent samples were randomly allocated to both groups according to the matching variables. The first author assessed the participants according to the inclusion criteria and allocated them into the groups.

### 2.3. Data Collection Tools

The data collection tool was a four-part questionnaire including demographic information, the sedation and analgesic checklist, visual analogue scale (VAS), and Verran and Snyder-Halpern sleep scale. The demographic questionnaire included age, sex, marital status, educational level, family income, smoking history, type of dialysis, date of operation, name of the surgeon, the duration of kidney failure, underlying diseases such as digestive diseases, diabetes, and hypertension, the patient's drugs (narcotics and sedation), and routine tests in the transplantation department such as hemoglobin, white blood cells, blood urea, platelets, creatinine, sodium, potassium, calcium, glucose, and liver enzymes. Sedation and analgesic checklist included midazolam, ketamine, fentanyl, cisatracurium, thiopental, lidocaine, morphine, and acetaminophen.

The visual analogue scale (VAS) was used to measure pain and fatigue [38]. The VAS is a self-administered visual scale that includes a 10 cm horizontal line graded from 0 to 10. Zero indicates no pain/fatigue, 1 – 3 indicates mild pain/fatigue, 4 – 7 indicates moderate pain/fatigue, and 8 – 10 indicates severe pain/fatigue. Many studies have confirmed the VAS reliability and validity [39, 40].

The Verran and Snyder-Halpern sleep scale was used to measure quality of sleep. This scale has 15 items that assess sleep in patients. This tool measures the participant's quality of sleep on the last night. It also includes various sleep parameters, such as sleep disrupters, number of waking cycles, difficulty in falling asleep, and sleep duration. The instrument is based on the scale value of 0 to 100 mm. The total score of the questionnaire is also between 0 and 100. The higher the score, the better the quality of sleep [41]. The reliability and validity of this scale have been confirmed in Iran [42].

### 2.4. Data Collection

The researchers referred to the research setting and started sampling after obtaining permission from the hospital management and the transplantation department. Demographic and background information was first assessed using the medical record and, if necessary, by asking the patients. Then, they were randomly divided into intervention and control groups according to the inclusion criteria. Pain, fatigue, and quality of sleep were measured on the first day of surgery to have the basis data. Pain, fatigue, and quality of sleep were measured on the second day after surgery before the intervention. We started the intervention on the second day to be sure that patients were in stable condition and attendance of the researchers in the ward did not interfere with the routine care. The intervention was conducted for three consecutive days. Again, pain, fatigue, and quality of sleep were measured immediately and one week after the intervention (i.e., the fourth day and eleventh day after surgery). The length of intervention was considered 3 sessions (daily) by reviewing the similar studies and consulting with the surgeon. In our center, patients are regularly discharged from the transplantation ward after 12–14 days. Therefore, we have chosen one-week follow-up.

The number of acetaminophen tablets (500 mg) taken by patients was also measured on the first, second, third, fourth, and eleventh days of surgery.

### 2.5. Intervention Protocol

The reflexology was applied to the intervention group according to the previous studies from the second day after surgery in the late hours of the evening shift and at least 4 hours after the last time when the patient received sedatives [30, 43, 44]. The evening shift was chosen because of the proximity to the patient's sleep hours and low workload of personnel. The patient's privacy was observed before the reflexology. The patient was placed in a relaxed position in a quiet and bright environment, and the researcher sat down on a chair at the bottom of the patient's bed and applied the reflexology. First, the researcher warmed her hands and cleaned the patient's feet with a warm wet napkin. Then, the feet were gently massaged for 3 minutes [33]. The researcher placed one hand at the back of big toe while applying pressure on the pituitary and pineal gland points. She took the heel of the foot with her left hand and applied pressure on the spinal points with the right-hand thumb [45]. She moved back and forth the patient's outer edge of the foot with her thumb [46]. The massage was applied with slow speed and regular rhythm with a tolerable pressure. The level of pressure was dependent on patients self-reporting of not feeling any pain regarding applying pressure. The massage of reflex zones lasted for nine minutes. Lastly, the foot was gently massaged for 3 minutes the same as the beginning of the procedure. Therefore, the protocol was performed for 15 minutes [34, 47] on each foot (30 minutes each session) in 3 sessions [48]. No lubricant was used for the application of the reflexology. It is noteworthy that the reflexology protocol was approved by the Iranian Art Massage Institute (https://artmassage.ir/). No reflexology was applied in the control group, and routine care of the transplantation department was taken. A nurse who did not know the assignment of the samples in the two groups completed patient's pain, fatigue, and quality of sleep scales by interviewing the participants immediately and one week after the intervention. It should be noted that the first researcher carried out all interventions. A Chinese medicine expert trained the first researcher in a 24-hour reflexology course. In addition, the first researcher received certification from the training center, i.e., the Iranian Art Massage Institute (https://artmassage.ir/).

### 2.6. Data Analysis

SPSS 18 was used to analyze the data. Independent *t*-test (or Mann–Whitney U test), chi-squared test, or Fisher's exact tests were used to determine the similarity of the two groups in terms of underlying and confounding variables at the beginning of the study. Repeated measures ANOVA or Friedman test was used to determine the mean difference in the pain, fatigue, and quality of sleep between the two groups.

### 2.7. Ethical Considerations

The ethics committee of Kerman University of Medical Sciences approved the protocol of the study(No.IR.KMU.REC.1397.071, IRCT20170116031972N6;https://en.irct.ir/trial/31687). The researchers explained research goals and protocol to the participants before their inclusion in the study, and if they had been willing to participate in the study, written informed consent would have been obtained from all eligible participants.

## 3. Results

The mean age of the participants in the foot reflexology group was 38.12 ± 12.87, and the mean age of them in the control group was 38.56 ± 12. The majority (81. 32%) of the samples in both groups were female. 60% of the samples in the foot reflexology group and 68% of the control group were married. 12% of the samples in both groups were uneducated. 48% of the samples in the foot reflexology group and 60% of the control group had diploma or more. 40% of the samples of both groups had family income less than a million toman (*P* > 0.05).

No significant difference was found between the two groups in the variables of sex, marital status, education level, and income level (*P* > 0.05). Furthermore, no significant difference was found between the two groups in clinical variables including duration of kidney failure (month), duration of dialysis (month), type of dialysis, history of diabetes, hypertension, heart diseases, and cigarette smoking (*P* > 0.05). No significant difference was observed between the two groups of foot reflexology and control in the results of laboratory tests before the kidney transplantation surgery (*P* > 0.05). One surgeon performed the surgery for the two groups, and they received similar medications during kidney transplantation. It should be noted that all surgical procedures lasted approximately 3 hours.

The mean pain score in the foot reflexology group decreased from 9.44 ± 0.96 on the day of surgery to 1.32 ± 0.94 on the eleventh day after surgery (*P* < 0.001). The mean pain score in the control group decreased from 9.36 ± 0.91 on the day of surgery to 4.32 ± 1.68 on the eleventh day after surgery (*P* < 0.001). Pain score significantly decreased three days after foot reflexology compared with the control group (*P* < 0.001). A significant decrease was found despite the completion of the intervention on the eleventh day after surgery (*P* < 0.001) (Table 1). No significant difference was found between the two groups in the use of acetaminophen on the first (*P* > 0.99), second (*χ*^2^ = 0.14, *P*=0.5), third (*χ*^2^ = 1.05, *P*=0.25), fourth (Fisher's exact test = 0.17, *P*=0.5), and eleventh days after surgery (Fisher's exact test = 0.34, *P*=0.5).

The mean fatigue score in the reflexology group decreased from 8.76 ± 1.27 on the day of surgery to 1.24 ± 1.2 on the eleventh day after surgery (*P* < 0.001). The mean fatigue score in the control group decreased from 8.6 ± 1.26 on the day of surgery to 3.92 ± 1.63 on the eleventh day after surgery (*P* < 0.001). Fatigue score significantly decreased three days after foot reflexology in comparison with the control group (*P* < 0.001). A significant decrease was seen despite the completion of intervention on the eleventh day after surgery (*P* < 0.001) (Table 2).

The mean sleep score in the foot reflexology group increased from 33.38 ± 11.22 on the day of surgery to 69.43 ± 12.8 on the eleventh day after surgery (*P* < 0.001). The mean sleep score in the control group increased from 39.59 ± 12.8 on the day of surgery to 56.27 ± 8.03 on the eleventh day after surgery (*P* < 0.001). The sleep score significantly increased three days after foot reflexology compared with the control group (*P*=0.01). Such an increase continued despite the completion of intervention on the eleventh day after surgery (*P* < 0.001) (Table 3).

In addition, no adverse effects were reported or observed during the intervention and follow-up period.

## 4. Discussion

The results of the present study showed that foot reflexology alleviated pain and fatigue and improved quality of sleep after kidney transplantation surgery. The effects of foot reflexology on pain, fatigue, and quality of sleep were clinically important. However, reflexology did not reduce acetaminophen consumption.

Few studies have investigated the effect of reflexology on pain of patients after kidney transplantation surgery. Most studies have examined reflexology effects on pain of patients after other surgeries. Other studies also confirmed the effect of reflexology on pain after abdominal hysterectomy [49], abdominal surgery [50], postoperative cancer cystectomy [51], abdominal and chest surgery [52], amputation of the lower extremity [53], and spinal surgery [30]. Koras and Karabulut found that although the pain intensity in patients undergoing laparoscopic cholecystectomy did not change for 5 minutes after reflexology, the intensity of pain reduced 30, 60, 90, and 120 minutes after reflexology [54]. Bhagya et al. reported that foot reflexology reduced pain of patients after open-heart surgery and sternotomy, cesarean section, and digestive system surgeries [29]. Mohammad Aliha et al. and Koras et al. also reported that foot reflexology reduced the consumption of analgesics and sedatives [52, 54]. These results do not support our findings. The difference in results may be because the patients in the study of Mohammad Aliha et al. were studied only during 24 hours after surgery and those in the study of Koras et al. were studied for 2 hours [52, 54]. However, in the current study, patients received the intervention from the second day after surgery and were examined for 11 days. Another reason for the inconsistency between our results and those of Mohammad Aliha et al. and Koras et al. [52, 54] may be the low rate of acetaminophen prescription in the present study. According to our data, only 4 to 28 percent of the participants took acetaminophen during their hospitalization after surgery and no other analgesics were prescribed for them. In general, most people struggle to deal with postoperative pain [20, 49]. Reflexology is assumed to increase secretion of endorphins and modulate pain impulse which positively affects pain perception [22]. Touch and massage of reflexology points, such as the pituitary, solar plexus, and spinal reflex zones, may reduce pain of patients after kidney transplantation.

The results of the current study showed severe fatigue in the foot reflexology group on the first and second days after the surgery, but the pain was moderate on the fourth day and mild on the eleventh day. The intensity of fatigue in the control group was severe on the first and second days after surgery, but it was moderate on the fourth and eleventh days. The intensity of fatigue significantly reduced three days after foot reflexology compared with the control group, and the effect of foot reflexology continued until the eleventh day after surgery. Few studies have investigated the effect of reflexology on fatigue after the kidney transplantation surgery or other surgeries [55], and no similar article was found on kidney transplanted patients. In the study of Mohammadi et al., the patients in the intervention group received a 30-minute foot reflexology on the second day after coronary artery bypass surgery (preferably the left foot). The results of the study showed reduction of fatigue in the intervention group 10 minutes after the intervention compared with the control group. However, the intensity of fatigue was not significantly different between the intervention and control groups 30 minutes and 24 hours after the intervention [55]. Mohammadi et al. do not support the results of the current study. It seems that the duration of the intervention and a different study population have influenced the treatment. Mohammadi et al. applied reflexology on patients only once, while the present study and Bagheri et al. applied reflexology on patients for three consecutive days. Furthermore, reflexology was applied on both feet of the patient in the current study while it was applied on the patient's foot in the study of Mohammadi et al. The index and thumb fingers are pressed on certain points of the foot that are associated with organs, glands, and other parts of the body. The blockage of energy in the body is assumed to be the cause of diseases, and reflexology massage of solar plexus can alleviate fatigue [44, 56].

The results of the current study showed the improvement of quality of sleep in both groups on the 4th and 11th days after surgery. The sleep score in the foot reflexology group increased by 36 at the end of the study, while the control group obtained nearly 17 points. A 19-point difference between the two groups shows the clinical importance of foot reflexology for the quality of sleep. Few studies have investigated the effect of reflexology on the quality of sleep of the patients after kidney transplantation surgery or other surgeries [26]. According to Kheyri et al., elderly women in the intervention group received foot reflexology twice a day (morning and night for 20 minutes) on the second and third days after abdominal surgery. The results of this study showed the improvement of quality of sleep in elderly women in the intervention group compared with the control group [26]. Several studies have also confirmed the positive effects of the foot reflexology on quality of sleep of the hemodialysis patients [28, 57]. Foot reflexology can stimulate the nervous system and provoke the dopamine secretion which may improve quality of sleep [58]. Since no research determined the effect of foot massage on quality of sleep after kidney transplantation surgery, further research is needed to confirm the effect of this intervention on these patients.

The present study has some limitations. Given that the transplantation rooms have two beds, in one case, one patient from the reflexology group and one patient from the control group were in one room adjacent to each other. However, a partition was used to reduce the effect of the intervention on the mentioned case in the control group. As the level of pressure was dependent on the patient tolerance, it was impossible to apply a uniform pressure for all patients. However, the level of pressure should naturally be adjusted with patient tolerance. In addition, the individual differences regarding the different perception of pain and fatigue are among the limitations of this research. The use of only one type of scale for measuring pain and fatigue can reduce the actual rate of perceived pain and fatigue. In addition, we used VAS for assessing fatigue. Although there are some more specific fatigue scales, we chose VAS as it was not time-consuming and was easy to understand in acute phase after kidney transplantation surgery. In addition, the first researcher allocated the samples into groups, which may be associated with bias. According to our literature review, this is the first study conducted on hospitalized patients under kidney transplantation surgery, and we adhered to minimum conditions of a randomized controlled trial. Therefore, the halo effect could not be rejected. Finally, the study setting was one of the referral transplantation centers in southeast Iran, so the results should be generalized with caution.

## 5. Conclusion

The results of the current study showed a significant reduction in the mean scores of pain and fatigue in the group of foot reflexology massage after the intervention compared with the control group. In addition, the quality of sleep score in the foot reflexology group significantly improved immediately and one week after the intervention compared with the control group. Therefore, foot reflexology may somewhat reduce pain and fatigue and improve sleep quality. Foot reflexology is a simple, low-cost, and applicable treatment that can be easily taught to nurses in various departments of the health care center. Given the limited evidence, further studies are needed to confirm the effects of foot reflexology on the symptoms after kidney transplantation surgery.

## Figures and Tables

**Table 1:** Comparison of mean score of pain in intervention and control groups.

	Group					
Pain	Intervention		Control		Mann–Whitney U test	*P* value
	Mean	SD	Mean	SD		
The first day of surgery	9.44	0.96	9.36	0.91	−0.64	0.52
Before intervention (the second day after surgery)	8.28	1.24	7.96	1.14	−1.15	0.25
Immediately after intervention (the fourth day after surgery)	3.48	1.26	5.72	1.7	−4.31	<0.001
One week after intervention (the eleventh day after surgery)	1.32	0.94	4.32	1.68	−5.53	<0.001
Friedman test	71.76		71.94		—	
*P* value	<0.001		<0.001		—	

**Table 2:** Comparison of mean score of fatigue in intervention and control groups.

	Group					
Fatigue	Intervention	Control			Statistical test ^*∗*^	*P* value	Mean difference	*P* value ^*∗∗*^
	Mean	SD	Mean	SD				
The first day of surgery	8.76	1.27	8.6	1.26	12.74	0.001	0.16	0.66
Before intervention (the second day after surgery)	7.52	1.16	7.2	1.68			0.32	0.44
Immediately after intervention (the fourth day after surgery)	3.32	1.41	5.56	1.66			−2.24	<0.001
One week after intervention (eleventh day after surgery)	1.24	1.2	3.92	1.63			−2.68	<0.001
Sphericity test	300.34		89.81		—			
*P* value	<0.001		<0.001		—			

^∗^Repeated measures analysis of variance. ^∗∗^Adjusted for multiple comparisons.

**Table 3:** Comparison of mean score of quality of sleep in intervention and control groups.

	Group					
Quality of sleep	Intervention	Control			Statistical test ^*∗*^	*P* value	Mean difference	*P* value ^*∗∗*^
	Mean	SD	Mean	SD				
The first day of surgery	33.38	11.22	39.59	12.8	10.84	<0.001	−6.21	0.07
Before intervention (the second day after surgery)	41.98	13.92	42.15	11.78			−0.17	0.96
Immediately after intervention (the fourth day after surgery)	60.60	10.75	52.23	11.76			8.38	0.01
One week after intervention (the eleventh day after surgery)	69.43	8.74	56.27	8.03			13.17	<0.001
Greenhouse–Geisser test	76.12		19.22		—			
*P* value	<0.001		<0.001		—			

^∗^Repeated measures analysis of variance. ^∗∗^Adjusted for multiple comparisons.

## Data Availability

The datasets used and/or analyzed during the current study are available from the corresponding author on reasonable request.

## Abbreviations

VAS: Visual analogue scale.
